# Crystal structure of di­chlorido­(4,11-dimethyl-1,4,8,11-tetra­aza­bicyclo­[6.6.2]hexa­deca­ne)iron(III) hexa­fluorido­phosphate

**DOI:** 10.1107/S2056989015015340

**Published:** 2015-08-26

**Authors:** Neil L. Funwie, Amy N. Cain, Brian Z. Fanning, Serena A. Hageman, Malorie Mullens, Travis K. Roberts, Daniel J. Turner, Cammi N. Valdez, Robert W. Vaughan, Henok G. Ermias, Jon D. Silversides, Stephen J. Archibald, Timothy J. Hubin, Timothy J. Prior

**Affiliations:** a Department of Chemistry and Physics, Southwestern Oklahoma State University, Weatherford, OK 73096, USA; b Department of Chemistry, University of Hull, Cottingham Road, Hull, HU6 7RX, England

**Keywords:** crystal structure, macrocycle, cross bridge, iron, cylam

## Abstract

The title compound, [FeCl_2_(C_14_H_30_N_4_)]PF_6_, contains Fe^3+^ coordinated by the four nitro­gen atoms of an ethyl­ene cross-bridged cyclam macrocycle and two *cis* chloride ligands in a distorted octa­hedral environment. In contrast to other similar compounds this is a monomer. Inter­molecular C—H⋯Cl inter­actions exist in the structure between the complex ions. Comparison with the mononuclear Fe^2+^ complex of the same ligand shows that the smaller Fe^3+^ ion is more fully engulfed by the cavity of the bicyclic ligand. Comparison with the μ-oxido dinuclear complex of an unsubstituted ligand of the same size demonstrates that the methyl groups of 4,11-dimethyl-1,4,8,11-tetra­aza­bicyclo­[6.6.2]hexa­decane prevent dimerization upon oxidation.

## Abstract 2

In contrast to other similar compounds, [FeCl_2_(C_14_H_30_N_4_)]PF_6_ is a monomer. Comparison with the mononuclear Fe^2+^ complex of the same ligand shows that the smaller Fe^3+^ ion is more fully engulfed by the cavity of the bicyclic ligand. Comparison with the μ-oxo dinuclear complex of an unsubstituted ligand of the same size demonstrates that the methyl groups of 4,11-dimethyl-1,4,8,11-tetra­aza­bicyclo­[6.6.2]hexa­decane prevent dimerization upon oxidation.

## Chemical context   

The tendency for iron complexes to form oxido-bridged iron(III) species and ultimately hydrated iron oxides limits their utility, especially in aqueous media, as functional catalysts based on common ligands (Ortiz de Montellano, 1986 ▸). Even so, iron is one of the predominant metal ions found in biological catalytic systems (Jang *et al.*, 1991 ▸; Wallar & Lipscomp, 1996 ▸; Boyington *et al.*, 1993 ▸). A major feature of numerous synthetic catalysts having nitro­gen donors and vacant coordination sites is their propensity to form dimers in which higher valent metal ions are present. One of us has produced iron(II) (Hubin *et al.*, 2000 ▸) and iron(III) (Hubin *et al.*, 2001 ▸) complexes of ethyl­ene cross-bridged tetra­aza­macrocyclic ligands that are remarkably resistant to oxidative hydrolysis while still having available sites for binding of the metal ion to either a terminal oxidant or substrate. The ability of the complex to remain mononuclear, and thus catalytically useful, appears to hinge on the substitution pattern of the non-bridgehead nitro­gen atoms of the bicyclic ligands (Hubin *et al.*, 2001 ▸). Methyl or benzyl substitution results only in mononuclear complexes, even in the *M* ^3+^ (Hubin *et al.*, 2001 ▸, 2003 ▸) or *M* ^4+^ (Yin *et al.*, 2006 ▸) oxidation state, while oxidation of the unsubstituted-ligand complexes results in μ-oxido iron(III) dimers (Hubin *et al.*, 2003 ▸). 

Recently, the iron(II) triflate complex of this same ligand, 4,11-dimethyl-1,4,8,11-tetra­aza­bicyclo­[6.6.2]hexa­decane, has been investigated as a catalyst for olefin oxidation by H_2_O_2_ and was found to be an active catalyst with reactivity properties similar to [Fe(TPA)(OTf)_2_] [TPA is tris(2-pyridyl­methyl)amine; OTf is trifluoromethanesulfonate; Feng *et al.*, 2011 ▸]. A key result of this study was that the location of two available *cis* binding sites on the metal ion is crucial for maximum catalytic activity. Very recently an Fe^IV^ analogue has been reported, but no crystal structure data were given (England *et al*., 2015 ▸).
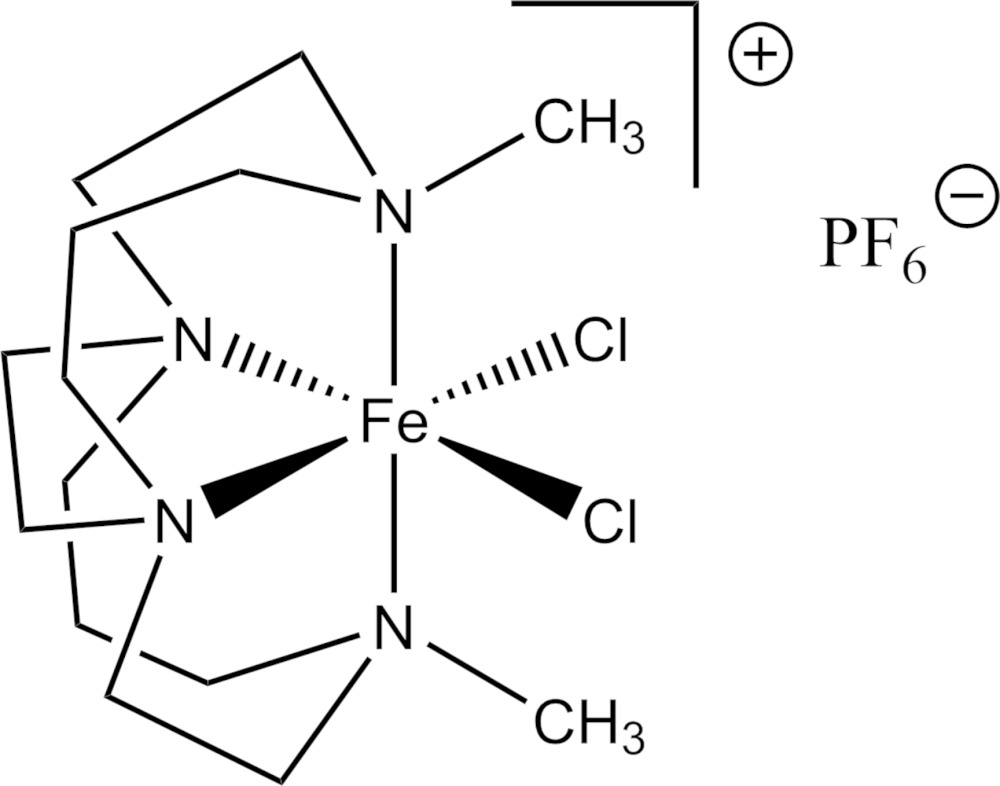


## Structural commentary   

**Figure 1:**
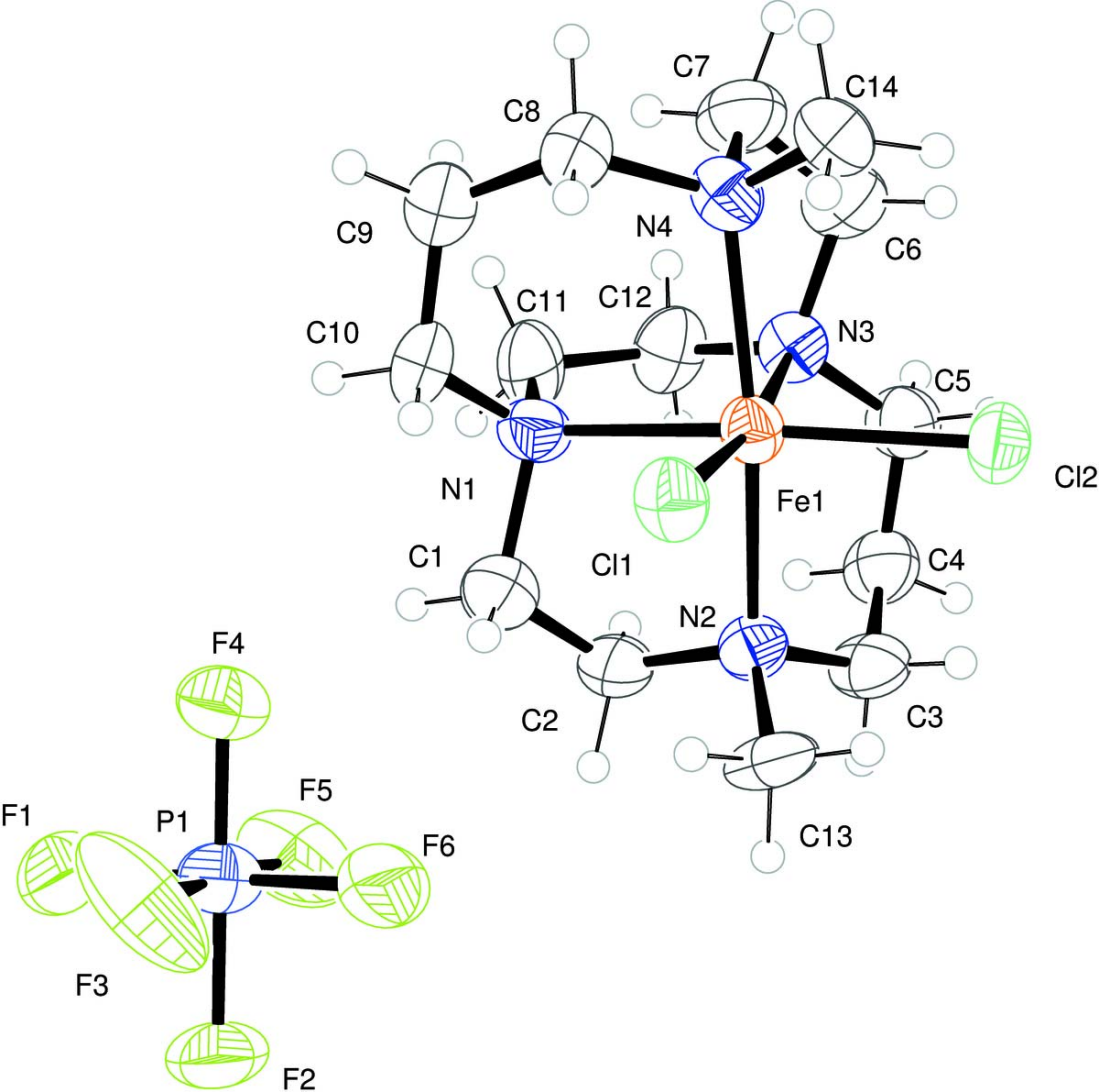
*ORTEP* representation of the asymmetric unit with atoms drawn as 50% probability ellipsoids.

The asymmetric unit of the title compound contains one complete Fe^3+^ mononuclear cross-bridged cyclam complex and a single PF_6_^−^ anion. The metal is hexa­coordinate in the so-called *cis*-V geometry common for macrocycles of this type. It is coordinated by four nitro­gen atoms of the macrocycle and two *cis* chloride ions, as shown in Fig. 1 ▸. The Fe—Cl bond lengths are similar to those of other comparable Fe^3+^ complexes. The relatively long Fe—N bonds strongly suggest the Fe^3+^ present is in a high-spin configuration.

The Fe^3+^ resides within a pocket in the rigid macrocycle, slightly displaced from the centre. The N2—Fe1—N4 bond angle is 166.8 (3) Å and the N1—Fe1—N3 bite angle is 79.8 (3) Å.

## Comparison with related structures   

Structural characterization of an Fe^3+^ mononuclear cross-bridged cyclam complex has not been achieved prior to the present study. In the present case, even upon oxidation of the iron from Fe(II) to Fe(III), the methyl-substituted ligand does not allow dimerization to occur. We will now compare the observed structure with that of the lower valent analogue and to the iron(III) μ-oxido dimer of the unsubstituted analogue.

**Figure 2:**
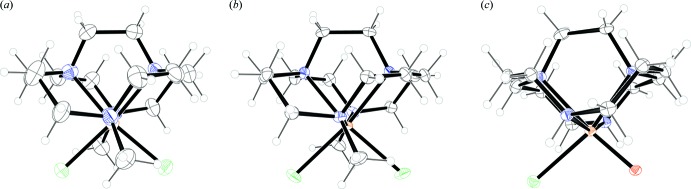
Comparison of Fe^2+^ complex (Hubin *et al.*, 2000 ▸) labelled (*a*), with Fe^3+^ monomer complex (*b*), and the one half of the dimer complex (Hubin *et al.*, 2003 ▸) (*c*), in each case viewed perpendicular to the Cl–Fe–Cl or Cl–Fe–O plane. Atoms are drawn as 50% probability ellipsoids.

**Figure 3:**
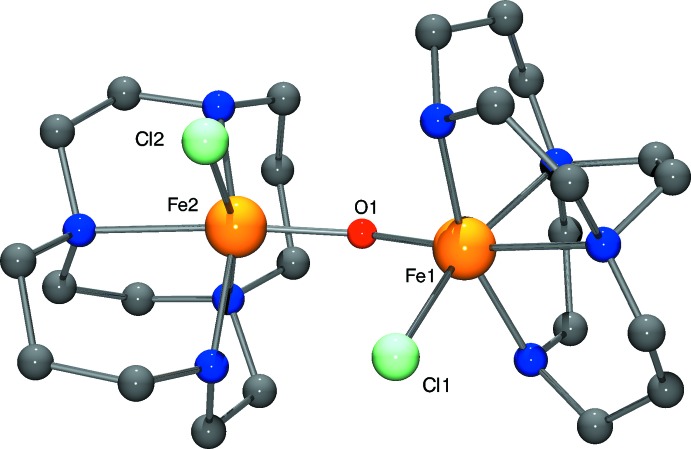
Mol­ecular structure of μ-oxidobis[chlorido­(1,4,8,11-tetra­aza­bicyclo[6.6.2]hexa­deca­ne)iron(III)] (Hubin *et al.*, 2003 ▸).

From a comparison of the Fe^3+^ 4,11-dimethyl-1,4,8,11-tetra­aza­bicyclo­[6.6.2]hexa­adecane dichloride complex hexa­fluorido­phosphate with the Fe^2+^ analogue, the reduction in ionic radius of the iron ion upon oxidation is clear (Table 1 ▸). N_ax_—Fe^3+^—N_ax_ is 166.8 (3)° in the present structure, while N_ax_—Fe^2+^—N_ax_ is 161.88 (5)° in the reduced complex (Hubin *et al.*, 2000 ▸). The smaller Fe^3+^ ion is pulled further into the ligand cavity as the favored octa­hedral geometry is approached, as can be seen by viewing each complex down the N_ax_—Fe^n+^—N_ax_ axis (Fig. 2 ▸). Fe—N bond lengths are also affected, going from a mean of 2.255 Å in the Fe^2+^ complex, to 2.209 Å in the Fe^3+^ complex. Comparison of the present Fe^3+^ monomer with the μ-oxido dimer complex is also informative. The crystal structure of the dimeric Fe^3+^ complex (Hubin, 2003 ▸) is represented in Fig. 3 ▸. The Fe^3+^ ion of this complex is also found in a pseudo-octa­hedral, six-coordinate geometry. Usually, these dimers contain five-coordinate metal cations, although dimers with six- and seven-coordinate cations are known (Murray, 1974 ▸). However, one monodentate chlorido ligand is maintained in this structure. Since the macrocyclic ligand is uncharged, the attractive Coulombic forces between the halide and the Fe^3+^ ion may be enough to keep it bound. Also, the folded ligand conformation helps separate the ligands from each other, easing the steric inter­actions that might favor lower coordination numbers with more nearly planar ligands. The secondary amine/Fe^3+^ bond lengths in the dimer are somewhat shorter than the tertiary amine/Fe^3+^ bond lengths: the Fe—N(secondary) mean length is 2.153 Å while the Fe—N(tertiary) mean length is 2.239 Å. In the monomer, with all tertiary amines, the mean Fe—N bond length is 2.209 Å, shorter but not quite matching that of the shortest, secondary amine bonds in the dimer. The N_ax_—Fe—N_ax_ mean bond angle is 161.6° in the dimer, while this value is 166.85 (19)° in the monomer. Clearly, dimerization, and its associated steric consequences, pulls the Fe^3+^ ion further out of the ligand cavity than it is in the Fe^3+^ monomer. In fact, the dimer N_ax_—Fe—N_ax_ bond angle is much closer to that of the Fe^2+^ monomer at 161.88 (5)° than that of the Fe^3+^ monomer at 166.85 (19)°. This steric consequence is consistent with the observation that the more sterically demanding methyl-substituted ligand prevents dimerization altogether.

## Supra­molecular features   

There are no classical hydrogen bonds within the structure, but many C—H⋯Cl and C—H⋯F inter­molecular inter­actions exist (Table 2 ▸). Pairs of complexes form dimers sustained by C—H⋯Cl inter­actions (H⋯Cl distances lie in the range 2.76 to 2.97 Å) and further C—H⋯Cl inter­actions link these into tapes that run parallel to the *b*-axis. These tapes are stacked along the *a* and *c* axes. Between them lie PF_6_^−^ anions, forming C—H⋯F inter­actions to generate a three-dimensional array of inter­molecular contacts.

## Database survey   

For coordination chemistry of cross-bridged tetra­aza­macrocycle derivatives and their applications, see: Hubin (2003 ▸); Jones *et al.* (2015 ▸); Springborg (2003 ▸); Wong *et al.* (2000 ▸). For related structures involving iron complexes of 4,11-dimethyl-1,4,8,11-tetra­aza­bicyclo­[6.6.2]hexa­decane derivatives, see: Hubin *et al.* (2000 ▸, 2001 ▸, 2003 ▸); McClain *et al.* (2006 ▸); Feng *et al.* (2011 ▸).

## Synthesis and crystallization   

The title complex was prepared by a procedure slightly modified from those found in Hubin *et al.* (2000 ▸, 2001 ▸). In an inert atmosphere glovebox, 0.381 g (0.001 mol) of the iron(II) dichloride complex of 4,11-dimethyl-1,4,8,11-tetra­aza­bicyclo­[6.6.2]hexa­decane (Hubin *et al.*, 2000 ▸) was dissolved in 20 ml of methanol in a round-bottom flask. Five equivalents of NH_4_PF_6_ (0.005 mol, 0.815 g) were dissolved in the solution. The flask was removed from the glovebox with a stopper to protect it from air. In a fume hood, a stream of nitro­gen gas was directed over the surface of the solution. Four to six drops of Br_2_ were added and the reaction was stirred for 15 minutes. Care must be taken when adding the bromine drops, as its vapor pressure and density tend to cause it to spurt out of the pipette. Practicing dispensing drops back into the bromine bottle (in the hood) can allow for successful dispensing.

A bright yellow–orange precipitate formed immediately. The nitro­gen gas was then allowed to bubble through the solution for 15 minutes to remove excess Br_2_. The flask was then stoppered and placed in a freezer for 30 minutes to complete the precipitation. The yellow–orange solid product was collected by vacuum filtration on a glass frit and washed with methanol and then ether. The product (0.428 g, 80% yield) was analytically pure as calculated with one-half molar equivalent of water of crystallization. Crystals suitable for X-ray diffraction were grown from ether diffusion into a di­chloro­methane solution.

## Refinement   

Standard data collection and refinement procedures were adopted. Crystal data, data collection and structure refinement details are summarized in Table 3 ▸.

Hydrogen atoms were placed using a riding model with fixed bond lengths and angles. For methyl­ene groups *U*_iso_ (H) was set at 1.2*U*_iso_(C) and for methyl groups *U*_iso_ (H) was set at 1.5*U*_iso_(C).

## Supplementary Material

Crystal structure: contains datablock(s) I. DOI: 10.1107/S2056989015015340/zl2640sup1.cif

Structure factors: contains datablock(s) I. DOI: 10.1107/S2056989015015340/zl2640Isup2.hkl

CCDC reference: 1419250

Additional supporting information:  crystallographic information; 3D view; checkCIF report

## Figures and Tables

**Table 1:** Geometric parameters (, ) for the macrocyclic cavity in Fe^2+^ and Fe^3+^ macrocycles*^*a*^*

Parameter	Fe^3+^Me_2_ *L^*b*^*	Fe^2+^Me_2_ *L^*c*^*	Fe^3+^H_2_ *L* dimer*^*d*^*
FeN1	2.195(5)	2.2574(13)	2.151
FeN2	2.229(5)	2.2866(14)	2.155
FeN3	2.220(5)	2.2634(13)	2.234
FeN4	2.190(5)	2.2748(13)	2.245
N2_ax_FeN4_ax_	166.8(3)	161.88(5)	161.6
N1_eq_FeN3_eq_	79.8(3)	78.36(5)	78.9

Notes: (*a*) where there are two independent molecules in the asymmetric unit, a mean value is given; (*b*) this work; (*c*) Hubin *et al.* (2000 ▸); (*d*) Hubin *et al.* (2003 ▸).

**Table 2:** Hydrogen-bond geometry (, )

*D*H*A*	*D*H	H*A*	*D* *A*	*D*H*A*
C1H1*A*F4	0.99	2.59	3.244(13)	124
C2H2*A*F1^i^	0.99	2.36	3.249(12)	148
C3H3*B*Cl2	0.99	2.73	3.304(11)	117
C5H5*B*Cl2	0.99	2.67	3.277(11)	120
C6H6*B*Cl2	0.99	2.91	3.453(10)	115
C8H8*A*Cl1	0.99	2.78	3.337(10)	116
C8H8*B*Cl2^ii^	0.99	2.78	3.726(11)	159
C9H9*A*Cl2^iii^	0.99	2.76	3.571(12)	140
C10H10*A*Cl1	0.99	2.69	3.311(11)	121
C11H11*A*F2^iv^	0.99	2.51	3.097(13)	118
C11H11*B*F5^i^	0.99	2.41	3.360(15)	161
C13H13*A*Cl1	0.98	2.63	3.198(10)	117
C13H13*B*F1^v^	0.98	2.59	3.323(11)	131
C14H14*A*Cl1	0.98	2.97	3.554(10)	119
C14H14*B*Cl1^vi^	0.98	2.89	3.772(11)	150
C14H14*B*Cl2	0.98	2.76	3.265(11)	113

Symmetry codes: (i) 
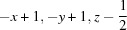
; (ii) 
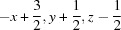
; (iii) 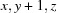; (iv) 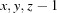; (v) 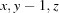; (vi) 
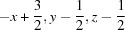
.

**Table 3:** Experimental details

Crystal data	
Chemical formula	[FeCl_2_(C_14_H_30_N_4_)]PF_6_
*M*_r_	526.14
Crystal system, space group	Orthorhombic, *P* *n* *a*2_1_
Temperature (K)	150
*a*, *b*, *c* ()	26.002(4), 8.5752(15), 9.3829(16)
*V* (^3^)	2092.1(6)
*Z*	4
Radiation type	Mo *K*
(mm^1^)	1.11
Crystal size (mm)	0.12 0.09 0.07

Data collection	
Diffractometer	Stoe IPDS2
No. of measured, independent and observed [*I* > 2(*I*)] reflections	21858, 4186, 2249
*R*_int_	0.143
(sin /)_max_ (^1^)	0.620

Refinement	
*R*[*F* ^2^ > 2(*F* ^2^)], *wR*(*F* ^2^), *S*	0.050, 0.109, 0.84
No. of reflections	4186
No. of parameters	254
No. of restraints	1
H-atom treatment	H-atom parameters constrained
_max_, _min_ (e ^3^)	0.39, 0.33
Absolute structure	Refined as a two-component inversion twin
Absolute structure parameter	0.03(5)

Computer programs: *X-AREA* (Stoe Cie, 2002 ▸), *SHELXS97* (Sheldrick, 2008 ▸), *SHELXL2014/7* (Sheldrick, 2015 ▸), *ORTEP-3 for Windows* (Farrugia, 2012 ▸) and *publCIF* (Westrip, 2010 ▸).

## supplementary crystallographic information

### Crystal data

**Table:**

[FeCl_2_(C_14_H_30_N_4_)]PF_6_	*D*_x_ = 1.670 Mg m^−^^3^
*M**_r_* = 526.14	Mo *K*α radiation, λ = 0.71073 Å
Orthorhombic, *P**n**a*2_1_	Cell parameters from 8849 reflections
*a* = 26.002 (4) Å	θ = 1.7–24.1°
*b* = 8.5752 (15) Å	µ = 1.11 mm^−^^1^
*c* = 9.3829 (16) Å	*T* = 150 K
*V* = 2092.1 (6) Å^3^	Block, orange
*Z* = 4	0.12 × 0.09 × 0.07 mm
*F*(000) = 1084	

### Data collection

**Table:**

Stoe IPDS2 diffractometer	2249 reflections with *I* > 2σ(*I*)
Radiation source: fine-focus sealed tube	*R*_int_ = 0.143
Detector resolution: 6.67 pixels mm^-1^	θ_max_ = 26.1°, θ_min_ = 1.6°
ω–scans	*h* = −32→32
21858 measured reflections	*k* = −10→8
4186 independent reflections	*l* = −11→11

### Refinement

**Table:**

Refinement on *F*^2^	Secondary atom site location: difference Fourier map
Least-squares matrix: full	Hydrogen site location: inferred from neighbouring sites
*R*[*F*^2^ > 2σ(*F*^2^)] = 0.050	H-atom parameters constrained
*wR*(*F*^2^) = 0.109	*w* = 1/[σ^2^(*F*_o_^2^) + (0.0378*P*)^2^] where *P* = (*F*_o_^2^ + 2*F*_c_^2^)/3
*S* = 0.84	(Δ/σ)_max_ = 0.001
4186 reflections	Δρ_max_ = 0.39 e Å^−^^3^
254 parameters	Δρ_min_ = −0.33 e Å^−^^3^
1 restraint	Absolute structure: Refined as a two-component inversion twin
Primary atom site location: structure-invariant direct methods	Absolute structure parameter: 0.03 (5)

### Special details

**Table:**

Geometry. All e.s.d.'s (except the e.s.d. in the dihedral angle between two l.s. planes) are estimated using the full covariance matrix. The cell e.s.d.'s are taken into account individually in the estimation of e.s.d.'s in distances, angles and torsion angles; correlations between e.s.d.'s in cell parameters are only used when they are defined by crystal symmetry. An approximate (isotropic) treatment of cell e.s.d.'s is used for estimating e.s.d.'s involving l.s. planes.
Refinement. Refined as a two-component inversion twin.

### Fractional atomic coordinates and isotropic or equivalent isotropic displacement parameters (Å^2^)

**Table:**

	*x*	*y*	*z*	*U*_iso_*/*U*_eq_	
Fe1	0.66232 (4)	0.14982 (13)	0.20386 (14)	0.0394 (3)	
Cl1	0.71279 (10)	0.2523 (4)	0.3803 (3)	0.0471 (6)	
Cl2	0.69285 (9)	−0.0987 (3)	0.2182 (3)	0.0493 (6)	
N1	0.6182 (3)	0.3664 (9)	0.1775 (7)	0.0479 (19)	
N2	0.5950 (3)	0.0889 (10)	0.3401 (7)	0.043 (2)	
N3	0.6144 (3)	0.0869 (10)	0.0162 (8)	0.042 (2)	
N4	0.7148 (3)	0.2294 (12)	0.0366 (8)	0.045 (2)	
C1	0.5844 (5)	0.3692 (15)	0.3148 (11)	0.058 (3)	
H1A	0.6066	0.3876	0.3990	0.069*	
H1B	0.5593	0.4558	0.3087	0.069*	
C2	0.5569 (4)	0.2208 (13)	0.3320 (11)	0.057 (3)	
H2A	0.5335	0.2044	0.2502	0.069*	
H2B	0.5360	0.2236	0.4201	0.069*	
C3	0.5698 (4)	−0.0588 (14)	0.2979 (10)	0.056 (3)	
H3A	0.5409	−0.0765	0.3645	0.067*	
H3B	0.5949	−0.1440	0.3138	0.067*	
C4	0.5490 (4)	−0.0768 (14)	0.1479 (10)	0.059 (3)	
H4A	0.5320	−0.1799	0.1407	0.071*	
H4B	0.5222	0.0036	0.1328	0.071*	
C5	0.5867 (4)	−0.0642 (13)	0.0324 (11)	0.055 (3)	
H5A	0.5689	−0.0866	−0.0585	0.066*	
H5B	0.6128	−0.1471	0.0463	0.066*	
C6	0.6538 (4)	0.0671 (13)	−0.0993 (10)	0.060 (3)	
H6A	0.6364	0.0548	−0.1924	0.072*	
H6B	0.6743	−0.0283	−0.0810	0.072*	
C7	0.6900 (5)	0.2105 (14)	−0.1046 (11)	0.067 (3)	
H7A	0.7166	0.1952	−0.1789	0.081*	
H7B	0.6701	0.3053	−0.1286	0.081*	
C8	0.7315 (4)	0.4001 (13)	0.0555 (11)	0.059 (3)	
H8A	0.7520	0.4078	0.1442	0.071*	
H8B	0.7545	0.4280	−0.0247	0.071*	
C9	0.6882 (5)	0.5214 (14)	0.0628 (13)	0.073 (4)	
H9A	0.7043	0.6258	0.0699	0.088*	
H9B	0.6693	0.5179	−0.0287	0.088*	
C10	0.6499 (4)	0.5070 (11)	0.1805 (13)	0.067 (3)	
H10A	0.6685	0.5103	0.2724	0.080*	
H10B	0.6269	0.5990	0.1773	0.080*	
C11	0.5873 (5)	0.3684 (14)	0.0505 (12)	0.064 (4)	
H11A	0.6044	0.4357	−0.0209	0.077*	
H11B	0.5537	0.4166	0.0735	0.077*	
C12	0.5775 (5)	0.2089 (12)	−0.0168 (11)	0.060 (3)	
H12A	0.5430	0.1728	0.0135	0.072*	
H12B	0.5765	0.2222	−0.1216	0.072*	
C13	0.6132 (4)	0.0676 (13)	0.4876 (9)	0.058 (3)	
H13A	0.6302	0.1632	0.5200	0.087*	
H13B	0.5838	0.0450	0.5497	0.087*	
H13C	0.6376	−0.0194	0.4911	0.087*	
C14	0.7641 (4)	0.1488 (14)	0.0423 (11)	0.058 (3)	
H14A	0.7795	0.1633	0.1366	0.088*	
H14B	0.7588	0.0373	0.0246	0.088*	
H14C	0.7871	0.1917	−0.0306	0.088*	
P1	0.58082 (9)	0.6220 (4)	0.6714 (2)	0.0514 (7)	
F1	0.5510 (2)	0.7847 (7)	0.6576 (6)	0.0737 (19)	
F2	0.5482 (3)	0.5900 (10)	0.8133 (7)	0.097 (3)	
F3	0.6235 (3)	0.7031 (11)	0.7626 (8)	0.117 (3)	
F4	0.6122 (2)	0.6531 (8)	0.5300 (6)	0.0635 (17)	
F5	0.5377 (3)	0.5381 (11)	0.5818 (8)	0.094 (3)	
F6	0.6097 (3)	0.4592 (7)	0.6862 (8)	0.094 (2)	

### Atomic displacement parameters (Å^2^)

**Table:**

	*U*^11^	*U*^22^	*U*^33^	*U*^12^	*U*^13^	*U*^23^
Fe1	0.0415 (6)	0.0379 (6)	0.0388 (6)	0.0011 (6)	−0.0025 (7)	−0.0031 (8)
Cl1	0.0505 (14)	0.0455 (15)	0.0452 (12)	−0.0004 (14)	−0.0107 (12)	−0.0074 (12)
Cl2	0.0602 (13)	0.0356 (11)	0.0522 (13)	0.0070 (10)	−0.0035 (13)	0.0016 (13)
N1	0.053 (4)	0.054 (5)	0.036 (5)	0.008 (4)	0.005 (4)	0.003 (4)
N2	0.048 (5)	0.045 (5)	0.037 (4)	0.002 (4)	−0.002 (4)	0.003 (4)
N3	0.039 (5)	0.045 (5)	0.042 (4)	−0.006 (4)	−0.009 (4)	−0.004 (4)
N4	0.041 (5)	0.048 (6)	0.047 (4)	−0.001 (5)	−0.002 (4)	−0.009 (4)
C1	0.066 (8)	0.055 (9)	0.052 (6)	−0.002 (7)	0.001 (5)	−0.006 (6)
C2	0.054 (7)	0.059 (8)	0.058 (7)	0.005 (6)	0.024 (5)	0.011 (6)
C3	0.061 (8)	0.056 (8)	0.049 (6)	−0.022 (6)	0.012 (5)	−0.002 (5)
C4	0.067 (7)	0.052 (7)	0.058 (6)	−0.012 (6)	−0.004 (6)	−0.002 (5)
C5	0.059 (7)	0.055 (8)	0.051 (6)	−0.007 (6)	0.002 (6)	−0.008 (5)
C6	0.072 (8)	0.067 (8)	0.040 (5)	−0.008 (6)	−0.009 (5)	0.000 (5)
C7	0.086 (8)	0.070 (8)	0.046 (6)	−0.011 (6)	0.007 (6)	0.004 (6)
C8	0.076 (8)	0.044 (7)	0.058 (6)	0.003 (6)	0.007 (5)	0.006 (5)
C9	0.086 (9)	0.047 (7)	0.086 (8)	0.002 (7)	0.032 (7)	0.002 (6)
C10	0.092 (8)	0.030 (5)	0.079 (9)	−0.005 (5)	0.005 (8)	0.007 (6)
C11	0.067 (9)	0.052 (9)	0.073 (7)	0.025 (7)	−0.008 (6)	0.003 (6)
C12	0.068 (7)	0.053 (8)	0.059 (6)	−0.004 (6)	−0.022 (6)	0.004 (6)
C13	0.071 (8)	0.068 (8)	0.035 (5)	−0.021 (6)	0.007 (5)	0.014 (5)
C14	0.053 (7)	0.059 (8)	0.064 (6)	0.010 (6)	0.013 (5)	−0.003 (6)
P1	0.0442 (14)	0.0654 (18)	0.0445 (16)	0.0126 (13)	0.0024 (11)	0.0086 (13)
F1	0.069 (4)	0.063 (4)	0.090 (5)	0.024 (3)	0.029 (3)	0.022 (3)
F2	0.123 (7)	0.094 (6)	0.075 (4)	0.050 (5)	0.058 (4)	0.038 (4)
F3	0.073 (5)	0.171 (9)	0.107 (5)	0.025 (5)	−0.027 (4)	−0.090 (5)
F4	0.060 (4)	0.077 (5)	0.054 (3)	0.004 (3)	0.012 (3)	0.000 (3)
F5	0.054 (4)	0.131 (8)	0.097 (5)	−0.021 (5)	0.017 (4)	−0.032 (5)
F6	0.123 (5)	0.081 (5)	0.079 (4)	0.046 (4)	0.038 (5)	0.019 (4)

### Geometric parameters (Å, º)

**Table:**

Fe1—N4	2.188 (8)	C6—C7	1.549 (14)
Fe1—N1	2.197 (7)	C6—H6A	0.9900
Fe1—N3	2.223 (7)	C6—H6B	0.9900
Fe1—N2	2.229 (8)	C7—H7A	0.9900
Fe1—Cl2	2.278 (3)	C7—H7B	0.9900
Fe1—Cl1	2.288 (3)	C8—C9	1.534 (15)
N1—C11	1.437 (13)	C8—H8A	0.9900
N1—C10	1.461 (11)	C8—H8B	0.9900
N1—C1	1.559 (12)	C9—C10	1.493 (15)
N2—C13	1.473 (12)	C9—H9A	0.9900
N2—C3	1.480 (14)	C9—H9B	0.9900
N2—C2	1.506 (13)	C10—H10A	0.9900
N3—C12	1.453 (13)	C10—H10B	0.9900
N3—C5	1.490 (13)	C11—C12	1.528 (15)
N3—C6	1.501 (12)	C11—H11A	0.9900
N4—C14	1.458 (12)	C11—H11B	0.9900
N4—C7	1.482 (13)	C12—H12A	0.9900
N4—C8	1.538 (15)	C12—H12B	0.9900
C1—C2	1.469 (16)	C13—H13A	0.9800
C1—H1A	0.9900	C13—H13B	0.9800
C1—H1B	0.9900	C13—H13C	0.9800
C2—H2A	0.9900	C14—H14A	0.9800
C2—H2B	0.9900	C14—H14B	0.9800
C3—C4	1.516 (13)	C14—H14C	0.9800
C3—H3A	0.9900	P1—F3	1.564 (7)
C3—H3B	0.9900	P1—F5	1.575 (8)
C4—C5	1.467 (14)	P1—F4	1.580 (6)
C4—H4A	0.9900	P1—F6	1.591 (6)
C4—H4B	0.9900	P1—F1	1.601 (6)
C5—H5A	0.9900	P1—F2	1.602 (7)
C5—H5B	0.9900		
			
N4—Fe1—N1	88.9 (3)	N3—C6—C7	110.3 (9)
N4—Fe1—N3	81.8 (3)	N3—C6—H6A	109.6
N1—Fe1—N3	79.8 (3)	C7—C6—H6A	109.6
N4—Fe1—N2	166.8 (3)	N3—C6—H6B	109.6
N1—Fe1—N2	81.5 (3)	C7—C6—H6B	109.6
N3—Fe1—N2	87.6 (3)	H6A—C6—H6B	108.1
N4—Fe1—Cl2	96.7 (3)	N4—C7—C6	108.8 (8)
N1—Fe1—Cl2	168.3 (2)	N4—C7—H7A	109.9
N3—Fe1—Cl2	90.9 (2)	C6—C7—H7A	109.9
N2—Fe1—Cl2	91.2 (2)	N4—C7—H7B	109.9
N4—Fe1—Cl1	92.4 (2)	C6—C7—H7B	109.9
N1—Fe1—Cl1	93.2 (2)	H7A—C7—H7B	108.3
N3—Fe1—Cl1	171.0 (2)	C9—C8—N4	116.3 (9)
N2—Fe1—Cl1	97.2 (2)	C9—C8—H8A	108.2
Cl2—Fe1—Cl1	96.70 (11)	N4—C8—H8A	108.2
C11—N1—C10	108.8 (9)	C9—C8—H8B	108.2
C11—N1—C1	111.8 (7)	N4—C8—H8B	108.2
C10—N1—C1	106.8 (8)	H8A—C8—H8B	107.4
C11—N1—Fe1	113.3 (6)	C10—C9—C8	117.9 (10)
C10—N1—Fe1	113.6 (6)	C10—C9—H9A	107.8
C1—N1—Fe1	102.3 (6)	C8—C9—H9A	107.8
C13—N2—C3	106.7 (8)	C10—C9—H9B	107.8
C13—N2—C2	110.6 (8)	C8—C9—H9B	107.8
C3—N2—C2	109.7 (8)	H9A—C9—H9B	107.2
C13—N2—Fe1	108.4 (6)	N1—C10—C9	115.5 (9)
C3—N2—Fe1	113.3 (6)	N1—C10—H10A	108.4
C2—N2—Fe1	108.2 (6)	C9—C10—H10A	108.4
C12—N3—C5	109.2 (8)	N1—C10—H10B	108.4
C12—N3—C6	112.3 (8)	C9—C10—H10B	108.4
C5—N3—C6	107.7 (8)	H10A—C10—H10B	107.5
C12—N3—Fe1	111.4 (6)	N1—C11—C12	115.2 (9)
C5—N3—Fe1	113.6 (6)	N1—C11—H11A	108.5
C6—N3—Fe1	102.5 (5)	C12—C11—H11A	108.5
C14—N4—C7	111.3 (8)	N1—C11—H11B	108.5
C14—N4—C8	101.4 (8)	C12—C11—H11B	108.5
C7—N4—C8	109.3 (8)	H11A—C11—H11B	107.5
C14—N4—Fe1	112.0 (6)	N3—C12—C11	116.5 (9)
C7—N4—Fe1	109.7 (6)	N3—C12—H12A	108.2
C8—N4—Fe1	113.0 (6)	C11—C12—H12A	108.2
C2—C1—N1	110.6 (9)	N3—C12—H12B	108.2
C2—C1—H1A	109.5	C11—C12—H12B	108.2
N1—C1—H1A	109.5	H12A—C12—H12B	107.3
C2—C1—H1B	109.5	N2—C13—H13A	109.5
N1—C1—H1B	109.5	N2—C13—H13B	109.5
H1A—C1—H1B	108.1	H13A—C13—H13B	109.5
C1—C2—N2	109.6 (9)	N2—C13—H13C	109.5
C1—C2—H2A	109.8	H13A—C13—H13C	109.5
N2—C2—H2A	109.8	H13B—C13—H13C	109.5
C1—C2—H2B	109.8	N4—C14—H14A	109.5
N2—C2—H2B	109.8	N4—C14—H14B	109.5
H2A—C2—H2B	108.2	H14A—C14—H14B	109.5
N2—C3—C4	119.6 (9)	N4—C14—H14C	109.5
N2—C3—H3A	107.4	H14A—C14—H14C	109.5
C4—C3—H3A	107.4	H14B—C14—H14C	109.5
N2—C3—H3B	107.4	F3—P1—F5	179.0 (5)
C4—C3—H3B	107.4	F3—P1—F4	91.0 (4)
H3A—C3—H3B	107.0	F5—P1—F4	89.8 (3)
C5—C4—C3	116.1 (9)	F3—P1—F6	90.5 (5)
C5—C4—H4A	108.3	F5—P1—F6	88.9 (5)
C3—C4—H4A	108.3	F4—P1—F6	88.7 (4)
C5—C4—H4B	108.3	F3—P1—F1	90.0 (4)
C3—C4—H4B	108.3	F5—P1—F1	90.6 (4)
H4A—C4—H4B	107.4	F4—P1—F1	92.0 (3)
C4—C5—N3	117.6 (9)	F6—P1—F1	179.2 (4)
C4—C5—H5A	107.9	F3—P1—F2	89.8 (5)
N3—C5—H5A	107.9	F5—P1—F2	89.4 (4)
C4—C5—H5B	107.9	F4—P1—F2	179.2 (4)
N3—C5—H5B	107.9	F6—P1—F2	91.5 (4)
H5A—C5—H5B	107.2	F1—P1—F2	87.8 (3)
			
C11—N1—C1—C2	69.7 (11)	C8—N4—C7—C6	−156.2 (9)
C10—N1—C1—C2	−171.5 (9)	Fe1—N4—C7—C6	−31.9 (11)
Fe1—N1—C1—C2	−51.9 (10)	N3—C6—C7—N4	57.8 (11)
N1—C1—C2—N2	58.7 (11)	C14—N4—C8—C9	−176.9 (9)
C13—N2—C2—C1	85.8 (10)	C7—N4—C8—C9	65.5 (11)
C3—N2—C2—C1	−156.8 (8)	Fe1—N4—C8—C9	−56.9 (10)
Fe1—N2—C2—C1	−32.7 (9)	N4—C8—C9—C10	60.6 (14)
C13—N2—C3—C4	−177.2 (10)	C11—N1—C10—C9	−63.7 (11)
C2—N2—C3—C4	63.0 (12)	C1—N1—C10—C9	175.6 (9)
Fe1—N2—C3—C4	−58.0 (12)	Fe1—N1—C10—C9	63.5 (11)
N2—C3—C4—C5	61.0 (15)	C8—C9—C10—N1	−64.2 (14)
C3—C4—C5—N3	−62.3 (14)	C10—N1—C11—C12	145.2 (10)
C12—N3—C5—C4	−62.7 (11)	C1—N1—C11—C12	−97.1 (11)
C6—N3—C5—C4	175.1 (9)	Fe1—N1—C11—C12	17.9 (12)
Fe1—N3—C5—C4	62.3 (11)	C5—N3—C12—C11	142.4 (10)
C12—N3—C6—C7	69.2 (10)	C6—N3—C12—C11	−98.1 (11)
C5—N3—C6—C7	−170.6 (8)	Fe1—N3—C12—C11	16.2 (12)
Fe1—N3—C6—C7	−50.5 (9)	N1—C11—C12—N3	−23.2 (15)
C14—N4—C7—C6	92.6 (11)		

### Hydrogen-bond geometry (Å, º)

**Table:**

*D*—H···*A*	*D*—H	H···*A*	*D*···*A*	*D*—H···*A*
C1—H1*A*···F4	0.99	2.59	3.244 (13)	124
C2—H2*A*···F1^i^	0.99	2.36	3.249 (12)	148
C3—H3*B*···Cl2	0.99	2.73	3.304 (11)	117
C5—H5*B*···Cl2	0.99	2.67	3.277 (11)	120
C6—H6*B*···Cl2	0.99	2.91	3.453 (10)	115
C8—H8*A*···Cl1	0.99	2.78	3.337 (10)	116
C8—H8*B*···Cl2^ii^	0.99	2.78	3.726 (11)	159
C9—H9*A*···Cl2^iii^	0.99	2.76	3.571 (12)	140
C10—H10*A*···Cl1	0.99	2.69	3.311 (11)	121
C11—H11*A*···F2^iv^	0.99	2.51	3.097 (13)	118
C11—H11*B*···F5^i^	0.99	2.41	3.360 (15)	161
C13—H13*A*···Cl1	0.98	2.63	3.198 (10)	117
C13—H13*B*···F1^v^	0.98	2.59	3.323 (11)	131
C14—H14*A*···Cl1	0.98	2.97	3.554 (10)	119
C14—H14*B*···Cl1^vi^	0.98	2.89	3.772 (11)	150
C14—H14*B*···Cl2	0.98	2.76	3.265 (11)	113

Symmetry codes: (i) −*x*+1, −*y*+1, *z*−1/2; (ii) −*x*+3/2, *y*+1/2, *z*−1/2; (iii) *x*, *y*+1, *z*; (iv) *x*, *y*, *z*−1; (v) *x*, *y*−1, *z*; (vi) −*x*+3/2, *y*−1/2, *z*−1/2.
